# Amino acid differences in glycoproteins B (gB), C (gC), H (gH) and L(gL) are associated with enhanced herpes simplex virus type-1 (McKrae) entry via the paired immunoglobulin-like type-2 receptor α

**DOI:** 10.1186/1743-422X-9-112

**Published:** 2012-06-13

**Authors:** Sona Chowdhury, Misagh Naderi, Vladimir N Chouljenko, Jason D Walker, Konstantin G Kousoulas

**Affiliations:** 1 Division of Biotechnology and Molecular Medicine and Department of Pathobiological Sciences, Louisiana State University School of Veterinary Medicine, Baton Rouge, LA 70803, USA

## Abstract

**Background:**

Herpes simplex virus type-1 (HSV-1) enters into cells via membrane fusion of the viral envelope with plasma or endosomal membranes mediated by viral glycoproteins. HSV-1 virions attach to cell surfaces by binding of viral glycoproteins gC, gD and gB to specific cellular receptors. Here we show that the human ocular and highly neurovirulent HSV-1 strain McKrae enters substantially more efficiently into cells via the gB-specific human paired immunoglobulin-like type-2 receptor-α (hPILR-α). Comparison of the predicted amino acid sequences between HSV-1(F) and McKrae strains indicates that amino acid changes within gB, gC, gH and gL may cause increased entry via the hPILR- α receptor.

**Results:**

HSV-1 (McKrae) entered substantially more efficiently than viral strain F in Chinese hamster ovary (CHO) cells expressing hPIRL-α but not within CHO-human nectin-1, -(CHO-hNectin-1), CHO-human HVEM (CHO-hHVEM) or Vero cells. The McKrae genes encoding viral glycoproteins gB, gC, gD, gH, gL, gK and the membrane protein UL20 were sequenced and their predicted amino acid (aa) sequences were compared with virulent strains F, H129, and the attenuated laboratory strain KOS. Most aa differences between McKrae and F were located at their gB amino termini known to bind with the PILRα receptor. These aa changes included a C10R change, also seen in the neurovirulent strain ANG, as well as redistribution and increase of proline residues. Comparison of gC aa sequences revealed multiple aa changes including an L132P change within the 129-247 aa region known to bind to heparan sulfate (HS) receptors. Two aa changes were located within the H1 domain of gH that binds gL. Multiple aa changes were located within the McKrae gL sequence, which were preserved in the H129 isolate, but differed for the F strain. Viral glycoproteins gD and gK and the membrane protein UL20 were conserved between McKrae and F strains.

**Conclusions:**

The results indicate that the observed entry phenotype of the McKrae strain is most likely due to a combination of increased binding to heparan sulfate receptors and enhanced virus entry via gB-mediated fusion of the viral envelope with plasma membranes.

## Introduction

Herpes simplex type 1 (HSV-1), Herpes simplex type 2 (HSV-2) and Varicella-zoster virus (VZV) are human neurotropic viruses that belong to the *Alphaherpesvirinae*subfamily and are a major cause of worldwide morbidity [1-4]. Neurovirulence, establishment of latency in sensory neurons and intermittent reactivation are some of the unique properties of these viruses [5,6]. Reactivation of latent virus from trigeminal ganglia can lead to recurrent ocular infections and is a leading cause of blindness in developed countries [7,8]. In very rare cases HSV-1 can spread spontaneously to the brain, causing life threatening herpes encephalitis [3].

Herpes virus initiates infection by binding to heparan sulfate (HS) moieties on cell surfaces using viral glycoproteins gC and gB [9]. Moreover, viral glycoprotein D (gD) binds to different cellular receptors including the herpesvirus entry mediator (HVEM, or HveA), nectin-1 (HveC), or 3-O-sulfated HS [10-12]. Apparently, gB can also bind to additional receptors including the paired immunoglobulin-like type 2 receptor alpha (PILRα), non-muscle myosin heavy chain IIA (NMHC-IIA), and myelin-associated glycoprotein (MAG) that function in virion attachment and virus entry [13-15]. HSV-1 enters into epithelial and neuronal cells via a pH-independent fusion of the viral envelope with plasma membranes, while it can enter into a wide range of non-neuronal cells via either pH-independent or pH-dependent endocytosis. Binding of gD and gB to their cognate receptors is thought to trigger sequential conformational changes in gH/gL and gB causing gB-mediated fusion of the viral envelope with cellular membranes during virus entry, as well as fusion among cellular membranes [16-18].

HSV-1 clinical isolates, such as the McKrae and H129 strains, are known to be highly virulent in rodents and rabbits in comparison to other laboratory strains such as KOS [19-21]. Several viral proteins and glycoproteins contribute to neurovirulence and latency in vivo, however their mode of action is not well elucidated [22-30]. HSV-1 gK is known to be involved in neurovirulence [30-34], and is a structural component of the virion particle functioning in virus entry into epithelial cells [35,36], cytoplasmic virion envelopment, virion egress and virus-induced cell fusion [37-39]. Recently, we showed that that HSV-1 gK and UL20 physically bind to gB and gH and modulate gB-mediated membrane fusion [40,41]. Also, we reported that gK is essential for virus spread in the cornea of mice, neuroinvasiveness and establishment of latency into ganglionic neurons [34].

In this manuscript, we compared the relative efficiency of virus entry between HSV-1 McKrae and F strains and found that McKrae entered substantially more efficiently into Chinese hamster ovary (CHO) cells expressing the hPIRLα. We sequenced all viral genes encoding viral glycoproteins involved in entry and cell-to-cell fusion and identified aa differences between McKrae and F strains that may cause the observed enhanced entry of McKrae over other viral strains.

## Results

**Figure 1:**
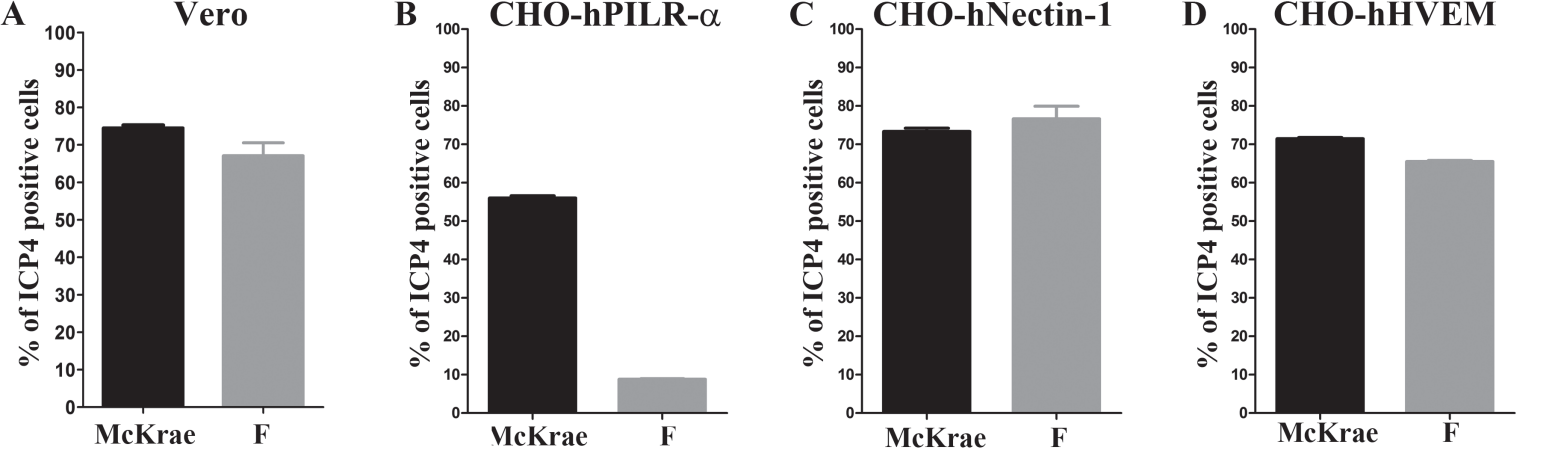
**Comparison of HSV-1(F) and McKrae entry efficiencies**. (A) Entry into Vero cells. (B) Entry into CHO-hPILRα. (C) Entry human CHO-nectin-1. (D) Entry into CHO-hHVEM (D). All cells were infected with HSV-1(F) or McKrae at an MOI of 1. At 12 h post-infection, the cells were stained with anti-ICP4 antibody and analyzed by flow cytometry to determine the percentage of infected cells.

**Figure 2:**
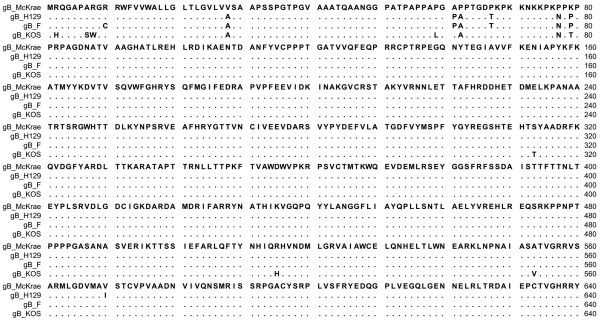
**Alignment of the predicted glycoprotein B (gB) amino acid sequences specified by HSV-1 strains McKrae, H129, F and KOS**. Amino acids that match the McKrae sequence are represented by dots. Amino acid changes that are different with respect to the HSV-1 McKrae strain are shown. The region of gB (aa 1-640), which contains aa substitutions is shown.

Both McKrae and F viruses appeared to enter with similar efficiency into Vero and CHO cells expressing either nectin-1 or HVEM (Figure 1: A, C, D, respectively). In contrast, F entered into CHO cells expressing PILRα substantially less efficiently than McKrae (Figure 1: B). Comparison of McKrae, F, KOS and H129 gB aa sequences (Figure 2) revealed that most of the aa differences between the four strains were located within the N-terminal 80 aa of gB. Amino acid comparison of four strains revealed that KOS had a number of aa that differed from the other HSV-1 strains. McKrae gB had a unique aa (A28V) within its predicted signal sequence (Figure 2). Comparison of F and McKrae gB sequences showed the following aa changes: C10R, A28V, P61A, A62P, T67P, N77P, and P79K (Table 1).

**Table 1:** HSV-1 (F) and McKrae gB Nucleotide and Amino acid Differences

Base#	F	McKrae	Amino acid #	Amino acid change
**28**	T	C	10	*Cys → Arg *
**83**	C	T	28	*Ala → Val*
**150**	A	C		
**181**	C	G	61	*Pro → Ala*
**184**	G	C	62	*Ala → Pro*
**199**	A	C	67	*Thr → pro*
**229**	A	C	77	*Asn → Pro*
**230**	A	C	77	*Asn → Pro*
**235**	C	A	79	*Pro → Lys*
**236**	C	A	79	*Pro → Lys*
**966**	T	C		
**1,513**	C	A		
**1,899**	C	T		
**1,932**	C	T		
**2,073**	C	A		
**2,196**	C	T		
**2,373**	T	C		

**Figure 3:**
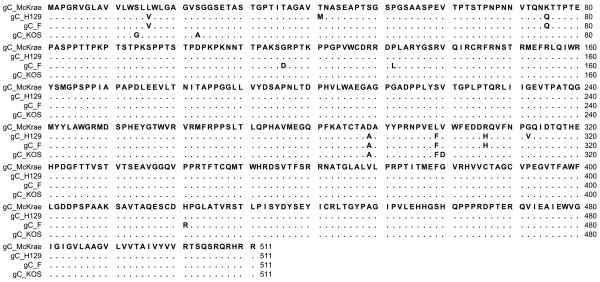
**Alignment of the predicted glycoprotein C (gC) amino acid sequences specified by HSV-1 strains McKrae, H129, F and KOS**. Amino acids that match the McKrae sequence are represented by dots. Amino acid changes that are different with respect to the HSV-1 McKrae strain are shown.

**Figure 4:**
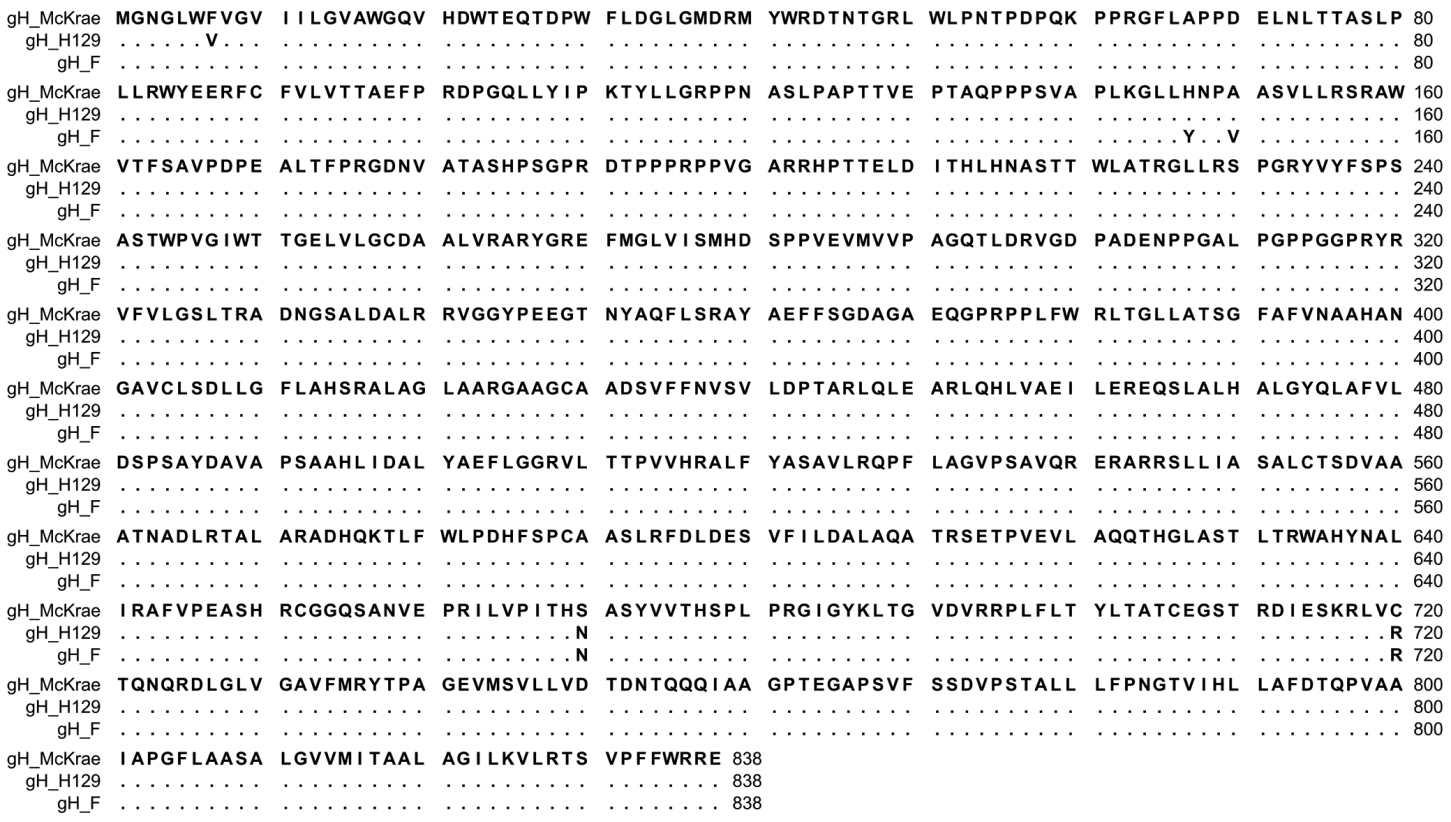
**Alignment of the predicted glycoprotein H (gH) amino acid sequences specified by HSV-1 strains McKrae, H129 and F**. Amino acids that match the McKrae sequence are represented by dots. Amino acid changes that are different with respect to the HSV-1 McKrae strain are shown.

**Figure 5:**
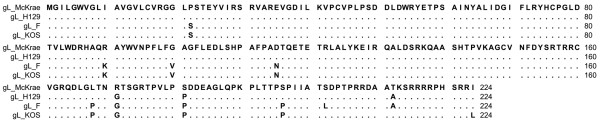
**Alignment of the predicted glycoprotein L (gL) amino acid sequences specified by HSV-1 strains McKrae, H129, F and KOS**. Amino acids that match the McKrae sequence are represented by dots. Amino acid changes that are different with respect to the HSV-1 McKrae strain are shown.

Comparison of the aa sequence of gC of HSV-1 McKrae, F, KOS and H129 revealed that aa variations between the four strains were distributed throughout the molecule (Figure 3). Although McKrae and KOS were more similar to each other, McKrae differed from all three strains at aa positions 289 (A289D) and 299(F299L). Specifically, there were 20 nucleotide differences between gC of F and McKrae strains. Out of 20 nucleotide differences only 8 resulted in the aa changes V16L, Q75K, D116G, L132P, A289D, F299L, H306R, R421H (Table 2). Nucleotide comparison between gD of HSV-1 F and McKrae strain revealed no aa differences between the McKrae and F strains (data not shown). Comparison of the McKrae, F and H129 gH aa sequences revealed that these proteins were highly conserved (Figure 4). There were only three aa differences in the H1 domain of gH and two aa differences within the C terminal H3 domain of gH. There were two unique aa substitutions (S670N and C720R) in McKrae gH that were not present in either strain F or H129. Moreover, HSV-1(F) and McKrae strains differed by four aa in gH (Y147H, V150A, N670S, and R720C) (Table 3). Amino acid alignment of gL encoded by McKrae, F, H129 and KOS revealed a number of aa differences that were spread across the entire molecule (Figure 5). The aa sequence of strain McKrae was similar to strain H129 except at aa residues 171,181 and 212. Comparison between nucleotide sequence of gL of HSV-1 F and McKrae strain revealed 19 nucleotide differences, ten of which coded for different aa (S22P, K90R, V100G, N115D, P168L, G171R, P181S, P196S, L202S, A212T) (Table 4). Seven of these aa changes were conserved in the H129 strain (P22, R90, G100, D115, L168, S196, S202) (Table 4). F and McKrae gK sequences were absolutely conserved despite four nucleotide changes within their genes. Similarly, there were four nucleotide changes between the UL20 gene of HSV-1 F and McKrae strains without causing any aa changes.

**Table 2:** HSV-1 (F) and McKrae gC Nucleotide and Amino acid Differences

Base#	F	McKrae	Amino acid #	Amino acid change
**46**	G	T	16	*Val → Leu*
**177**	G	A		
**223**	C	A	75	*Gln → Lys*
**279**	A	G		
**347**	A	G	116	*Asp → Gly*
**393**	T	C		
**394**	T	C	132	*Leu → Pro*
**395**	T	C		
**594**	A	G		
**619**	G	A		
**636**	C	T		
**866**	C	A	289	*Ala → Asp*
**897**	T	G	299	*Phe → Leu*
**917**	A	G	306	*His → Arg*
**981**	A	C		
**1,071**	G	A		
**1,254**	G	A		
**1,262**	G	A	421	*Arg → His*
**1,365**	G	A		
**1,485**	A	G		

**Table 3:** HSV-1 (F) and McKrae gH Nucleotide and Amino acid Differences

Base#	F	McKrae	Amino acid #	Amino acid change
**439**	T	C	147	*Tyr → His*
**449**	T	C	150	*Val → Ala*
**558**	A	G		
**1,596**	G	T		
**1,650**	A	C		
**1,899**	C	T		
**2,009**	A	G	670	*Asn → Ser*
**2,067**	C	G		
**2,158**	C	T	720	*Arg → Cys*
**2,217**	A	G		
**2,301**	G	A		

**Table 4:** HSV-1 (F) and McKrae gL Nucleotide and Amino acid Differences

Base#	F	McKrae	Amino acid #	Amino acid change
**21**	C	T		
**64**	T	C	22	*Ser → Pro*
**165**	T	C		
**261**	T	C		
**269**	A	G	90	*Lys → Arg*
**273**	A	G		
**294**	A	G		
**299**	T	G	100	*Val → Gly*
**327**	C	T		
**333**	A	G		
**343**	A	G	115	*Asn → Asp*
**489**	A	C		
**503**	C	T	168	*Pro → Leu*
**511**	G	A	171	*Gly → Arg*
**541**	C	T	181	*Pro → Ser*
**586**	C	T	196	*Pro → Ser*
**605**	T	C	202	*Leu → Ser*
**634**	G	A	212	*Ala → Thr*
**660**	C	T		

## Discussion

HSV-1 utilizes multiple receptors to attach and enter into a variety of cells including neurons. Recently, it was shown that gB binds to cellular receptors that are required for gB-mediated membrane fusion during virus entry and virus-induced cell fusion. We show here that the HSV-1 McKrae strain utilizes the gB receptor PIRLα more efficienty than HSV-1(F). DNA sequencing of all viral glycoprotein genes involved in membrane fusion indicates that there are a number of aa differences between HSV-1(F) and McKrae in gB, gH and gL that may affect PIRLα- mediated virus entry.

Initial entry experiments revealed that PIRL-α did not function as efficiently in facilitating virion entry in comparison to the gD receptors nectin-1 and HVEM when strains HSV-1(F) and KOS were used (not shown). Recently, we cloned the HSV-1(McKrae) strain as a bacterial articifical chromosome (bac) that has enabled us to rapidly produce mutant viruses, as we have previously done with HSV-1(F) bac [42,43]. We have found that this viral strain efficiently enters into a variety of cells including CHO cells constitutively expressing PIRLα. PIRLα binds gB and membrane fusion can be affected by interactions of gB with viral glycoproteins gD, gH/gL and gK/UL20 [18,40,41]. Therefore, we compared the predicted aa sequences between F and McKrae strains and found a number of aa that could contribute to the observed increased efficiency of entry of McKrae over F and other strains. Of particular importance were aa changes between F and McKrae located within the amino terminus of gB, known to bind to PIRLα and gK [41,44]. Specifically, gB binds to PIRLα via O-linked glycans located at aa positions 53 and 480 [44]. However, gB binding to PIRLα is also dependent on the conformation of the amino terminus of gB, since aa insertions in gB could reduce this binding [45]. Insertional mutagenesis has revealed that the structure and function of gB is not particularly flexible in tolerating aa insertions [46]. The McKrae gB contains additional proline residues at aa positions 67 and 77, while other proline residues have been re-arranged. Specifically, the F gB has a proline at aa 61, which is an alanine for the McKrae strain, but the McKrae gB contains a proline at aa 62 instead of alanine. The structure of the amino terminus of gB is not known, although the x-ray structure of gB (aa 111-726) was obtained [17]. The additional proline residues suggest that the amino terminus of gB assumes a conformation that may affect binding to PIRLα [44,47-49]. In addition, this altered conformation of gB may affect interactions with gK, which binds to the amino terminus of gB and regulates gB-mediated membrane fusion [41]. Interestingly, six aa changes seen in McKrae versus F gB were conserved in the gB specified by the neurovirulent strain ANG [22] suggesting that these aa may contribute to neurovirulence.

Viral glycoproteins gD and gH have been shown to bind gB and modulate its ability to cause membrane fusion [18]. Therefore, mutations within the extracellular portions of gD and gH, as well as gL may affect the ability of gB to utilize the PIRL-α receptor. In addition, since gH forms a functional heterodimer with gL [50], it is possible that the observed aa differences between McKrae and F within gH domain H1 may affect interaction with gL, known to bind exclusively to this domain [51]. The carboxyl terminus of gH has been shown to be important for virus-induced cell fusion [43]. Therefore, the observed aa changes N670S and R720C may alter virus entry kinetics. Multiple aa changes in gL between McKrae and F (S22P, K90R, V100G, N115D) are within the gL domain known to interact with gH and may affect gH/gL cell-surface expression, cell fusion and virus entry.

Positively charged aa residues are known to be critical for interaction with negatively charged sulfate/carboxylate groups of the HS chain [52]. Moreover, basic aa residues are known to be critical for HS-binding activity [53]. Therefore, the gC Q75K and H306R aa changes in McKrae compared to F may cause increased HS binding. Previous studies have delineated the HS-binding domain that interacts with gC amino terminal residues to be located between aa 33 and 123 [54], and between 129 and 247 [55]. Therefore amino acid changes in McKrae gC (aa 75, 116 and 132) may result in increased initial binding of McKrae gC.

The PILR alpha gene is expressed on cells of the immune system (monocytes, dendritic cells, NK cells, B cells, macrophages, neutrophils, eosinophils, mast cells), as well as neurons [13,56]. Moreover, HSV-1 enters into corneal epithelial cells (HCE) via the nectin1, HVEM and PILRα receptors [57]. Additional experiments are needed to determine the biological and pathogenetic implications of increased utilization of the PIRLα receptor by the HSV-1(McKrae). The availability of the McKrae strain as a bacterial artificial chromosome will enable the rapid construction of mutant viruses that could be used to elucidate the role of each viral glycoprotein in PIRLα mediated virion entry.

## Materials and methods

### Cells and Viruses

The clinical ocular isolate and neuroinvasive strain of HSV-1 (the parental wild-type), McKrae strain, was obtained from Dr. J. M. Hill (Louisiana State University Health Sciences Center, New Orleans, LA, USA). African green monkey kidney (Vero) cells were obtained from the American Type Culture Collection (Rockville, MD, USA) and grown and propagated in Dulbecco's modified Eagle Medium (DMEM) supplemented with 7% fetal bovine serum (FBS) and antibiotics. The HSV-1 McKrae strain was maintained as a low passage stock on Vero cells. CHO-neo cells and CHO-nectin 1 were a kind gift from Dr. Yasushi Kawaguchi, (The University of Tokyo, Tokyo, Japan) and were propagated in Ham's F12 medium supplemented with 10% fetal calf serum (FCS) and 250 μg G418/ml. The CHO-hPLIRα cells were obtained from Dr. Hishashi Arase, (Osaka University, Osaka Japan), were grown in Ham's F12 medium supplemented with 10% fetal calf serum (FCS) and puromycin, 1 ug/ml. The human CHO-HVEM was created in our laboratory using PiggyBac Transposon system (System Biosciences). HVEM gene was cloned into PB514BL-1 PiggyBac Dual Promoter Vector and CHO cells expressing HVEM were selected using puromycin, 12 ug/ml. All cells were cultured in non-selective medium prior to use in infectivity assays.

### Viral DNA extraction and DNA sequencing

Viral DNA was isolated from infected Vero cells as described earlier for tissue samples [34]. Briefly, confluent monolayers of Vero cells were infected at a multiplicity of infection of 2 and harvested by scraping at 24 hour post-infection. Cell pellets were rinsed with PBS and viral DNA was extracted using the DNAeasy Blood and Tissue Kit according to manufacturer's instruction (Qiagen, Valencia, CA, USA).

Purified McKrae DNA and Fail Safe DNA polymerase (Epicentre Biotechnologies, Madison, WI) were used for PCR. Due to the high GC content of the genomic DNA, PCR was performed using a series of primers which generated overlapping products encompassing the entire gene to be sequenced. PCR products were column-purified (Zymo Research Corp., Orange, CA) and sequencing reactions were prepared using the Big Dye Terminator v3.1 Cycle sequencing Kit (Life Technologies). DNA sequencing was performed in both directions by a primer walking strategy for each gene using an automated DNA sequencer (3130 Genetic Analyzer, Applied Biosystems). The list of all synthetic oligo-nucleotides used for amplification of the McKrae genes and sequencing is shown in the Additional file 1, Table 1.

### Sequence assembly and nucleotide and amino acid alignments

The Sequencher (4.10.1) software package was used to assemble the overlapping fragments using default parameters. Sequence assembly was performed using the HSV-1 (F) strain as the reference sequence (Gene Bank:GU734771.1). Nucleotide sequences of the following McKrae genes: gB, gC, gD, gH, gL, gK and UL20 were submitted to GenBank (accession numbers: JQ320080.1, JQ359758.1, JQ320083.1, JQ320081.1, JQ359759.1, JQ320082.1, JQ320079.1).

The CLC sequence Viewer version 6 software was used to align the aa sequences of H129 (GenBank: GU734772.1), F (GenBank: GU734771.1) and KOS strain (gB:AAG34116.1; gC: AAA45779.1; gL: AAA99790.1) against the derived McKrae sequences. Alignment was performed using the default parameters of the Clustal W alignment program (CLC sequence viewer software). To determine nucleotide and aa sequence variation between the McKrae and F strains, the sequences were aligned by the MegAlign program in the DNASTAR software package (Lasergene). The differences in nucleotides and aa in the coding region of each sequenced gene were tabulated in Tables 1, 2, 3 and 4.

### Virus entry assays

Confluent monolayers of CHO-neo, CHO-hHVEM, CHO-hPILRα, CHO-human nectin-1 and Vero cells were infected with HSV-1 strains (McKrae and F) at a multiplicity of infection of 1 for 1 hr at 34°C. The virus inoculum was subsequently removed, and the cultures were shifted to 37°C. Twelve hours post-infection (hpi), the cells were fixed and stained with anti-ICP4 antibody (Virusys, Inc., Taneytown, MD) and Alexa Fluor 647 goat anti-mouse IgG1(Life Technologies, Grand Island, NY). The relative efficiency of virus entry was calculated by flow cytometry as the percentage of cells expressing ICP4 normalized to the CHO-neo entry values. Mean values and standard deviations of three independent experiments were calculated.

## Abbreviations

HVEM, Herpes virus entry mediator; CHO, Chinese hamster ovary; hPILRα, Human paired immunoglobulin-like type 2 receptor α

## Competing interests

The authors declare that they have no competing interests.

## Authors' contributions

SC was primarily responsible for virus entry experiments, sequence assembly of all genes and interpretation of amino acid differences. MN performed DNA sequencing reactions and assisted in virus entry experiments and gene sequence assembly. JW was responsible for designing the flow cytometry experiments. VC and KK were responsible for overall interpretation of the results and manuscript preparation. All authors read and approved the final manuscript. 

## Supplementary Material

Additional file 1 **Table S1**. Sequencing and PCR primers. Click here for file

## References

[B1] Fisman DN Lipsitch M Hook EW Goldie SJ Projection of the future dimensions and costs of the genital herpes simplex type 2 epidemic in the United States Sex Transm Dis 2002 29 608 622 10.1097/00007435-200210000-00008 12370529

[B2] Stahl JP Mailles A Dacheux L Morand P Epidemiology of viral encephalitis in 2011 Med Mal Infect 2011 41 453 464 10.1016/j.medmal.2011.05.015 21802875

[B3] Steiner I Herpes simplex virus encephalitis: new infection or reactivation? Curr Opin Neurol 2011 24 268 274 10.1097/WCO.0b013e328346be6f 21483260

[B4] Zamora MR DNA viruses (CMV, EBV, and the herpesviruses) Semin Respir Crit Care Med 2011 32 454 470 10.1055/s-0031-1283285 21858750

[B5] Whitley RJ Kimberlin DW Roizman B Herpes simplex viruses Clin Infect Dis 1998 26 541 553 quiz 554-545 10.1086/514600 9524821

[B6] Whitley R Knipe D, Howley P Herpes Simplex Viruses Fields Virology 2001 Philadelphia, PA: Lippincott Williams and Wilkins 2461 2510

[B7] Liesegang TJ Melton LJ Daly PJ Ilstrup DM Epidemiology of ocular herpes simplex. Incidence in Rochester, Minn, 1950 through 1982 Arch Ophthalmol 1989 107 1155 1159 10.1001/archopht.1989.01070020221029 2787981

[B8] Liesegang TJ Herpes simplex virus epidemiology and ocular importance Cornea 2001 20 1 13 10.1097/00003226-200101000-00001 11188989

[B9] Herold BC WuDunn D Soltys N Spear PG Glycoprotein C of herpes simplex virus type 1 plays a principal role in the adsorption of virus to cells and in infectivity J Virol 1991 65 1090 1098 1847438 10.1128/jvi.65.3.1090-1098.1991 PMC239874

[B10] Geraghty RJ Krummenacher C Cohen GH Eisenberg RJ Spear PG Entry of alphaherpesviruses mediated by poliovirus receptor-related protein 1 and poliovirus receptor Science 1998 280 1618 1620 10.1126/science.280.5369.1618 9616127

[B11] Montgomery RI Warner MS Lum BJ Spear PG Herpes simplex virus-1 entry into cells mediated by a novel member of the TNF/NGF receptor family Cell 1996 87 427 436 10.1016/S0092-8674(00)81363-X 8898196

[B12] Shukla D Liu J Blaiklock P Shworak NW Bai X Esko JD Cohen GH Eisenberg RJ Rosenberg RD Spear PG A novel role for 3-O-sulfated heparan sulfate in herpes simplex virus 1 entry Cell 1999 99 13 22 10.1016/S0092-8674(00)80058-6 10520990

[B13] Satoh T Arii J Suenaga T Wang J Kogure A Uehori J Arase N Shiratori I Tanaka S Kawaguchi Y PILRalpha is a herpes simplex virus-1 entry coreceptor that associates with glycoprotein B Cell 2008 132 935 944 10.1016/j.cell.2008.01.043 18358807 PMC2394663

[B14] Arii J Goto H Suenaga T Oyama M Kozuka-Hata H Imai T Minowa A Akashi H Arase H Kawaoka Y Kawaguchi Y Non-muscle myosin IIA is a functional entry receptor for herpes simplex virus-1 Nature 2010 467 859 862 10.1038/nature09420 20944748

[B15] Suenaga T Satoh T Somboonthum P Kawaguchi Y Mori Y Arase H Myelin-associated glycoprotein mediates membrane fusion and entry of neurotropic herpesviruses Proc Natl Acad Sc USA 2010 107 866 871 10.1073/pnas.0913351107 20080767 PMC2818916

[B16] Hannah BP Heldwein EE Bender FC Cohen GH Eisenberg RJ Mutational evidence of internal fusion loops in herpes simplex virus glycoprotein B J Virol 2007 81 4858 4865 10.1128/JVI.02755-06 17314168 PMC1900191

[B17] Heldwein EE Lou H Bender FC Cohen GH Eisenberg RJ Harrison SC Crystal structure of glycoprotein B from herpes simplex virus 1 Science 2006 313 217 220 10.1126/science.1126548 16840698

[B18] Connolly SA Jackson JO Jardetzky TS Longnecker R Fusing structure and function: a structural view of the herpesvirus entry machinery Nat Rev Microbiol 2011 9 369 381 10.1038/nrmicro2548 21478902 PMC3242325

[B19] Hill JM Rayfield MA Haruta Y Strain specificity of spontaneous and adrenergically induced HSV-1 ocular reactivation in latently infected rabbits Curr Eye Res 1987 6 91 97 10.3109/02713688709020074 3030660

[B20] Hill TJ Ocular pathogenicity of herpes simplex virus Curr Eye Res 1987 6 1 7 10.3109/02713688709020060 3030632

[B21] Perng GC Mott KR Osorio N Yukht A Salina S Nguyen QH Nesburn AB Wechsler SL Herpes simplex virus type 1 mutants containing the KOS strain ICP34.5 gene in place of the McKrae ICP34.5 gene have McKrae-like spontaneous reactivation but non-McKrae-like virulence J Gen Virol 2002 83 2933 2942 12466469 10.1099/0022-1317-83-12-2933

[B22] Kosovsky J Vojvodova A Oravcova I Kudelova M Matis J Rajcani J Herpes simplex virus 1 (HSV-1) strain HSZP glycoprotein B gene: comparison of mutations among strains differing in virulence Virus Genes 2000 20 27 33 10.1023/A:1008104006007 10766304

[B23] Cameron JM McDougall I Marsden HS Preston VG Ryan DM Subak-Sharpe JH Ribonucleotide reductase encoded by herpes simplex virus is a determinant of the pathogenicity of the virus in mice and a valid antiviral target J Gen Virol 1988 69 Pt 10 2607 2612 2844969 10.1099/0022-1317-69-10-2607

[B24] Gordon YJ Simon PL Armstrong JA Neurovirulence of an herpes simplex type 1 thymidine kinase negative mutant determined by virus biochemical defect and host immune system in mice. Brief report Arch Virol 1984 80 225 229 10.1007/BF01310662 6326711

[B25] Kurachi R Daikoku T Tsurumi T Maeno K Nishiyama Y Kurata T The pathogenicity of a US3 protein kinase-deficient mutant of herpes simplex virus type 2 in mice Arch Virol 1993 133 259 273 10.1007/BF01313767 8257288

[B26] Nishiyama Y Kurachi R Daikoku T Umene K The US 9, 10, 11, and 12 genes of herpes simplex virus type 1 are of no importance for its neurovirulence and latency in mice Virology 1993 194 419 423 10.1006/viro.1993.1279 8386887

[B27] Perng GC Thompson RL Sawtell NM Taylor WE Slanina SM Ghiasi H Kaiwar R Nesburn AB Wechsler SL An avirulent ICP34.5 deletion mutant of herpes simplex virus type 1 is capable of in vivo spontaneous reactivation J Virol 1995 69 3033 3041 7707530 10.1128/jvi.69.5.3033-3041.1995 PMC189003

[B28] Imai T Arii J Minowa A Kakimoto A Koyanagi N Kato A Kawaguchi Y Role of the herpes simplex virus 1 Us3 kinase phosphorylation site and endocytosis motifs in the intracellular transport and neurovirulence of envelope glycoprotein B J Virol 2011 85 5003 5015 10.1128/JVI.02314-10 21389132 PMC3126194

[B29] Yuhasz SA Stevens JG Glycoprotein B is a specific determinant of herpes simplex virus type 1 neuroinvasiveness J Virol 1993 67 5948 5954 8396662 10.1128/jvi.67.10.5948-5954.1993 PMC238015

[B30] Rajcani J Kudelova M Glycoprotein K of herpes simplex virus: a transmembrane protein encoded by the UL53 gene which regulates membrane fusion Virus Genes 1999 18 81 90 10.1023/A:1008025520655 10334040

[B31] Mott KR Chentoufi AA Carpenter D BenMohamed L Wechsler SL Ghiasi H The role of a glycoprotein K (gK) CD8+ T-cell epitope of herpes simplex virus on virus replication and pathogenicity Invest Ophthalmol Vis Sci 2009 50 2903 2912 10.1167/iovs.08-2957 19168902

[B32] Mott KR Perng GC Osorio Y Kousoulas KG Ghiasi H A recombinant herpes simplex virus type 1 expressing two additional copies of gK is more pathogenic than wild-type virus in two different strains of mice J Virol 2007 81 12962 12972 10.1128/JVI.01442-07 17898051 PMC2169076

[B33] Osorio Y Mott KR Jabbar AM Moreno A Foster TP Kousoulas KG Ghiasi H Epitope mapping of HSV-1 glycoprotein K (gK) reveals a T cell epitope located within the signal domain of gK Virus Res 2007 128 71 80 10.1016/j.virusres.2007.04.007 17499382 PMC2020453

[B34] David AT Baghian A Foster TP Chouljenko VN Kousoulas KG The herpes simplex virus type 1 (HSV-1) glycoprotein K(gK) is essential for viral corneal spread and neuroinvasiveness Curr Eye Res 2008 33 455 467 10.1080/02713680802130362 18568883

[B35] Foster TP Rybachuk GV Kousoulas KG Glycoprotein K specified by herpes simplex virus type 1 is expressed on virions as a Golgi complex-dependent glycosylated species and functions in virion entry J Virol 2001 75 12431 12438 10.1128/JVI.75.24.12431-12438.2001 11711633 PMC116139

[B36] Jambunathan N Chowdhury S Subramanian R Chouljenko VN Walker JD Kousoulas KG Site-specific proteolytic cleavage of the amino terminus of herpes simplex virus glycoprotein K on virion particles inhibits virus entry J Virol 2011 85 12910 12918 10.1128/JVI.06268-11 21994443 PMC3233161

[B37] Hutchinson L Roop-Beauchamp C Johnson DC Herpes simplex virus glycoprotein K is known to influence fusion of infected cells, yet is not on the cell surface J Virol 1995 69 4556 4563 7769723 10.1128/jvi.69.7.4556-4563.1995 PMC189205

[B38] Foster TP Kousoulas KG Genetic analysis of the role of herpes simplex virus type 1 glycoprotein K in infectious virus production and egress J Virol 1999 73 8457 8468 10482598 10.1128/jvi.73.10.8457-8468.1999 PMC112865

[B39] Jayachandra S Baghian A Kousoulas KG Herpes simplex virus type 1 glycoprotein K is not essential for infectious virus production in actively replicating cells but is required for efficient envelopment and translocation of infectious virions from the cytoplasm to the extracellular space J Virol 1997 71 5012 5024 9188566 10.1128/jvi.71.7.5012-5024.1997 PMC191734

[B40] Chouljenko VN Iyer AV Chowdhury S Chouljenko DV Kousoulas KG The amino terminus of herpes simplex virus type 1 glycoprotein K (gK) modulates gB-mediated virus-induced cell fusion and virion egress J Virol 2009 83 12301 12313 10.1128/JVI.01329-09 19793812 PMC2786757

[B41] Chouljenko VN Iyer AV Chowdhury S Kim J Kousoulas KG The herpes simplex virus type 1 UL20 protein and the amino terminus of glycoprotein K (gK) physically interact with gB J Virol 2010 84 8596 8606 10.1128/JVI.00298-10 20573833 PMC2919038

[B42] Melancon JM Fulmer PA Kousoulas KG The herpes simplex virus UL20 protein functions in glycoprotein K (gK) intracellular transport and virus-induced cell fusion are independent of UL20 functions in cytoplasmic virion envelopment Virol J 2007 4 120 10.1186/1743-422X-4-120 17996071 PMC2186317

[B43] Melancon JM Luna RE Foster TP Kousoulas KG Herpes simplex virus type 1 gK is required for gB-mediated virus-induced cell fusion, while neither gB and gK nor gB and UL20p function redundantly in virion de-envelopment J Virol 2005 79 299 313 10.1128/JVI.79.1.299-313.2005 15596825 PMC538735

[B44] Wang J Fan Q Satoh T Arii J Lanier LL Spear PG Kawaguchi Y Arase H Binding of herpes simplex virus glycoprotein B (gB) to paired immunoglobulin-like type 2 receptor alpha depends on specific sialylated O-linked glycans on gB J Virol 2009 83 13042 13045 10.1128/JVI.00792-09 19812165 PMC2786847

[B45] Fan Q Lin E Satoh T Arase H Spear PG Differential effects on cell fusion activity of mutations in herpes simplex virus 1 glycoprotein B (gB) dependent on whether a gD receptor or a gB receptor is overexpressed J Virol 2009 83 7384 7390 10.1128/JVI.00087-09 19457990 PMC2708615

[B46] Lin E Spear PG Random linker-insertion mutagenesis to identify functional domains of herpes simplex virus type 1 glycoprotein B Proc Natl Acad Sci USA 2007 104 13140 13145 10.1073/pnas.0705926104 17666526 PMC1941792

[B47] Tabata S Kuroki K Wang J Kajikawa M Shiratori I Kohda D Arase H Maenaka K Biophysical characterization of O-glycosylated CD99 recognition by paired Ig-like type 2 receptors J Biol Chem 2008 283 8893 8901 10.1074/jbc.M709793200 18234675

[B48] Wang J Shiratori I Satoh T Lanier LL Arase H An essential role of sialylated O-linked sugar chains in the recognition of mouse CD99 by paired Ig-like type 2 receptor (PILR) J Immunol 2008 180 1686 1693 18209065 10.4049/jimmunol.180.3.1686 PMC2577149

[B49] Williamson MP The structure and function of proline-rich regions in proteins Biochem J 1994 297 Pt 2 249 260 8297327 10.1042/bj2970249 PMC1137821

[B50] Fan Q Lin E Spear PG Insertional mutations in herpes simplex virus type 1 gL identify functional domains for association with gH and for membrane fusion J Virol 2009 83 11607 11615 10.1128/JVI.01369-09 19726507 PMC2772692

[B51] Chowdary TK Cairns TM Atanasiu D Cohen GH Eisenberg RJ Heldwein EE Crystal structure of the conserved herpesvirus fusion regulator complex gH-gL Nat Struct Mol Biol 2010 17 882 888 10.1038/nsmb.1837 20601960 PMC2921994

[B52] Cardin AD Weintraub HJ Molecular modeling of protein-glycosaminoglycan interactions Arteriosclerosis 1989 9 21 32 10.1161/01.ATV.9.1.21 2463827

[B53] Trybala E Roth A Johansson M Liljeqvist JA Rekabdar E Larm O Bergstrom T Glycosaminoglycan-binding ability is a feature of wild-type strains of herpes simplex virus type 1 Virology 2002 302 413 419 10.1006/viro.2002.1639 12441085

[B54] Tal-Singer R Peng C Ponce De Leon M Abrams WR Banfield BW Tufaro F Cohen GH Eisenberg RJ Interaction of herpes simplex virus glycoprotein gC with mammalian cell surface molecules J Virol 1995 69 4471 4483 7769707 10.1128/jvi.69.7.4471-4483.1995 PMC189189

[B55] Trybala E Bergstrom T Svennerholm B Jeansson S Glorioso JC Olofsson S Localization of a functional site on herpes simplex virus type 1 glycoprotein C involved in binding to cell surface heparan sulphate J Gen Virol 1994 75 Pt 4 743 752 7512117 10.1099/0022-1317-75-4-743

[B56] Fan Q Longnecker R The Ig-like v-type domain of paired Ig-like type 2 receptor alpha is critical for herpes simplex virus type 1-mediated membrane fusion J Virol 2010 84 8664 8672 10.1128/JVI.01039-10 20573830 PMC2919009

[B57] Shah A Farooq AV Tiwari V Kim MJ Shukla D HSV-1 infection of human corneal epithelial cells: receptor-mediated entry and trends of re-infection Mol Vis 2010 16 2476 2486 21139972 PMC2994737

