# Supplementary material for: Amino acid differences in glycoproteins B (gB), C (gC), H (gH) and L(gL) are associated with enhanced herpes simplex virus type-1 (McKrae) entry via the paired immunoglobulin-like type-2 receptor α
Source: Virol J. 2012 Jun 13;9:112. doi: 10.1186/1743-422X-9-112 (PMC3402990; doi:10.1186/1743-422X-9-112)
Supplement: Additional file 1 — Table S1. Sequencing and PCR primers. [file 1743-422X-9-112-S1.DOCX]

**Table S1.** Sequencing and PCR primers

| Primer name | Primer sequence |
| --- | --- |
| gB1 | 5’-tctcgatcctctactcggtcc-3’ |
| gB2 | 5’-gcagaccgacgggcgctttggcac-3’ |
| gB3 | 5’-ggaggaggtggtcttgatgcg-3’ |
| gB4 | 5’-gccccttctcagcaacacgctcgc-3’ |
| gB7 | 5’-ataccaaataagacccattggtgtatgttc-3’ |
| gB8 | 5’-agaaccccaccaacccggacgcgt-3’ |
| gB9 | 5’-tcgcaggtgtggttcggccac-3’ |
| gB10 | 5’-tctgcaccatgaccaagtggcag-3’ |
| gB11 | 5’-tggtcttcgtaccgaaagctgac-3’ |
| gB12 | 5’-ttggacatgaaggaggacacg-3’ |
|  |  |
| gC1 | 5’-tgtggaggtcgttttcatgc-3’ |
| gC2 | 5’-ggtgacagaatacaacggagggt-3’ |
| gC3 | 5’-agaggaggtcctgacgaacatcac-3’ |
| gC4 | 5’-aggatgacctgagggaagagag-3’ |
| gC5 | 5’-agtttgtctggttcgaggacgac-3’ |
| gC6 | 5’-aggtcacggtagagacggtggtg-3’ |
|  |  |
| gD1 | 5’-taagcttcagcgcgaacgaccaactac-3’ |
| gD2 | 5’-cgctaccgacttatcgactgtccgcctt-3’ |
| gD3 | 5’-agattacacagtttatcctggagca-3’ |
| gD4 | 5’-atctccgtccagtcgtttatc-3’ |
|  |  |
| gH1 | 5’-tcgcgccagtacatgcggtccatg-3’ |
| gH2 | 5’-gcaagccctgccatagcc-3’ |
| gH3 | 5’-ggaccaccatcacttccacc-3’ |
| gH4 | 5’-agctggacatcacgcacctgcac-3’ |
| gH5 | 5’-tggagccgagcctctagctgcag-3’ |
| gH6 | 5’-atccgtggctcgacgttggcag-3’ |
| gH7 | 5’-tgtcctccggcagccgttcttgg-3’ |
| gH8 | 5’-aacaaaccacagacacggttacag-3’ |
| gH9 | 5’-tgatccgcgccttcgtccctg-3’ |
| gH10 | 5’-agcctagcaggtcggagaggcag-3’ |
| gH11 | 5’-tctgcctctccgacctgctag-3’ |
|  |  |
| gK1 | 5’-caacaaccgcctgtgcgtgtc-3’ |
| gK2 | 5’-tgggatccaggctacacgtgg-3’ |
| gK3 | 5’-actcacgacaccaaacatacatc-3’ |
| gK4 | 5’-tgctctgcgagctgtcggtccag-3’ |
|  |  |
| gL1 | 5’-acgacgtcgtggttgtgttactg-3’ |
| gL2 | 5’-acgaggaaaacgtcacaccag-3’ |
|  |  |
| UL20 F | 5’-gttatatcaaggcgcggaac-3’ |
| UL20 R | 5’-tcgttccaggagaacccagtgt-3’ |
